# Effects of Plant Polysaccharides Combined with Boric Acid on Digestive Function, Immune Function, Harmful Gas and Heavy Metal Contents in Faeces of Fatteners

**DOI:** 10.3390/ani14111515

**Published:** 2024-05-21

**Authors:** Juan Deng, Feng Zhang, Haoran Fan, Yuxuan Zheng, Chunfang Zhao, Man Ren, Erhui Jin, Youfang Gu

**Affiliations:** 1 College of Animal Science, Anhui Science and Technology University, Chuzhou 233100, China; w1754068518@163.com (J.D.); zhangfeng@ahstu.edu.cn (F.Z.); 19810611712@163.com (H.F.); zyx19980306@163.com (Y.Z.); zhaocf@ahstu.edu.cn (C.Z.); renm@ahstu.edu.cn (M.R.); 2 Anhui Province Key Laboratory of Animal Nutrition Regulation and Health, Chuzhou 233100, China

**Keywords:** plant polysaccharides, boric acid, fatteners, digestive enzymes, immune, antioxidant, harmful gases

## Abstract

**Simple Summary:**

With the transformation of the breeding industry towards a healthy breeding model, the application of green feed additives in pig production is gradually increasing. Many plant polysaccharides are widely used as safe and green additives in animal production. Boron may be a necessary trace element for animals and its application in animal husbandry is also increasing. The study on the application effect of the combined addition of plant polysaccharides and boron in fatteners production performance, immune function, antioxidant function, digestive function and harmful substance emissions is of great significance for the rational use of both. This study added boron and different types of plant polysaccharides to the diet in the form of boric acid and found that the combined addition of boric acid and plant polysaccharides can improve the production performance and antioxidant function of fatteners by enhancing the activity of intestinal digestive enzymes and reducing the content of harmful substances such as heavy metals and harmful gases in feces and urine, thereby effectively improving healthy growth and reducing environmental pollution today.

**Abstract:**

The experiment aimed to investigate the effects of plant polysaccharides combined with boric acid on digestive function, immune function and harmful gas and heavy metal contents in the faeces of fatteners. For this study, 90 healthy crossbred fatteners were selected and randomly divided into five groups: the control group was fed with a basal diet (Con); experimental group I was fed with basal diet + 40 mg/kg boric acid (BA); experimental group II was fed with basal diet + 40 mg/kg boric acid + 400 mg/kg *Astragalus* polysaccharides (BA+APS); experimental group III was fed with basal diet + 40 mg/kg boric acid + 200 mg/kg *Ganoderma lucidum* polysaccharides (BA+GLP); and experimental group IV was fed with basal diet + 40 mg/kg boric acid + 500 mg/kg *Echinacea* polysaccharides (BA+EPS). Compared with Con, the average daily gain (ADG), the trypsin activities in the duodenum and jejunum, the IL-2 levels in the spleen, the T-AOC activities and GSH-Px contents in the lymph node of fattening were increased in the BA group (*p* < 0.05), but malondialdehyde content in the lymph and spleen, and the contents of NH_3_, H_2_S, Hg, Cu, Fe and Zn in the feces and urine were decreased (*p* < 0.05). Compared with the BA, the ADG, gain-to-feed ratio (G/F), the trypsin and maltase activities in the duodenum and jejunum were increased in the BA+APS (*p* < 0.05), and the T-SOD activities in the spleen and T-AOC activities in the lymph node were also increased (*p* < 0.05), but the H_2_S level was decreased in the feces and urine (*p* < 0.05). Compared with the BA, the ADG, G/F and the trypsin and maltase activities in the duodenum were increased in the BA+GLP and BA+EPS (*p* < 0.05), the activities of maltase and lipase in the duodenum of fatteners in the BA+GLP and the activities of trypsin, maltase and lipase in the BA+EPS were increased (*p* < 0.05). Gathering everything together, our findings reveal that the combined addition of boric acid and plant polysaccharides in the diet of fatteners synergistically improved their growth performance and immune status. That may be achieved by regulating the activity of intestinal digestive enzymes, improving the antioxidant function and then promoting the digestion and absorption of nutrients. Furthermore, the above results reduce the emission of harmful gases and heavy metals in feces and urine.

## 1. Introduction

Antibiotics, as a drug that can inhibit and kill bacteria, are widely used in the livestock and poultry industry, playing a crucial role in preventing and treating animal diseases and promoting animal growth. Research has confirmed that the unreasonable use of veterinary antibiotics can lead to increased resistance to pathogenic bacteria, increased drug residues in animal products, decreased meat and egg quality and intensified environmental pollution [1]. In this situation, many countries have issued “anti-antibiotic orders” to reduce the application of antibiotics in animal husbandry production. Antibiotic substitution has gradually become a hot topic of concern. Under the trend of healthy and green development in animal husbandry, the utilization of antibiotic alternatives has become crucial for sustainable development in the field of livestock husbandry [2]. More and more literature reports that some organic trace elements, medicinal plants and fungi and their extracts can be ideal alternatives to antibiotics. This is because most medicinal plants, fungi and their extracts not only have a significant immune-enhancing effect but also have a certain promoting effect on animal growth. Many organic trace elements not only have antioxidant and immune-enhancing effects but also have the effect of improving the quality of meat and eggs [3]. In addition, medicinal plants, fungi and their extracts share common advantages with organic trace elements, such as low toxicity, no residue and no drug resistance [4]. Therefore, the application of medicinal plants, their extracts and organic trace elements in the healthy breeding of livestock and poultry is becoming increasingly widespread.

Plant and fungal polysaccharides are common plant or fungi extracts that are the main active ingredient for plants and fungi to exert physiological regulatory effects on animal bodies. *Astragalus* membranaceus, *Echinacea* purpurea and *Ganoderma lucidum* are common medicinal plants and fungi. Their extracts (polysaccharides) have broad biological activities, which can enhance the antioxidant and immune functions of animal bodies, improve digestive enzyme activity, promote animal cell growth and improve production performance. Research has found that adding 800 mg/kg *Astragalus* polysaccharides to the diet can increase the average daily weight gain and feed conversion rate of LPS-infected weaned piglets, promote cytokine expression, enhance antioxidant enzyme activity and thereby improve immune and digestive functions [5,6]. There are also reports pointing out that the addition of *Echinacea* at an appropriate level can enhance macrophage bactericidal ability, thereby regulating host defense against pathogen invasion and enhancing immune function in mice [7]. 

Boric acid is a weak inorganic acid and its boron element is an essential mineral element in animal bodies. Research has found that, like many organic trace elements, boron has lower toxicity to animal bodies. Supplementing boron elements in the form of boric acid in an appropriate amount can improve gastrointestinal pH and intestinal tissue structure and regulate intestinal microbiota and pepsin activity, thereby promoting nutrient digestibility [8]. In addition, boric acid can improve growth performance and enhance immunity and antioxidant function, while reducing the release of toxic and harmful substances from the body [9]. Furthermore, the results of previous experiments conducted by our lab have revealed that the addition of appropriate amounts of boric acid could mediate the apoptosis and immune function response of splenic lymphocytes in rats, indicating that boric acid could be added in appropriate amounts as an immunomodulatory agent in animal foods [10,11,12]. 

With the continuous improvement of people’s living standards, countries around the world are paying more and more attention to environmental pollution. The production of manure and harmful gases from pig farms is one of the main sources of environmental pollution, which not only has adverse effects on the healthy growth environment of animals but also poses a threat to human health [13]. How to effectively reduce environmental pollution caused by pig farming is gradually becoming a hot research topic. Research has found that adding green feed additives can improve animal digestive function, increase nutrient utilization and feed conversion rates, effectively reduce the emission of toxic and harmful substances in feces and urine, and thereby reduce environmental pollution, adding non-starch polysaccharides to the diet can improve the absorption rate of nutrients by fatteners, effectively control fecal excretion, reduce CO_2_ and other harmful gas emissions and further reduce environmental pollution [14,15].

However, as an ideal alternative to antibiotics and beneficial trace elements, it is still unclear whether *Astragalus* polysaccharides, *Echinacea* polysaccharides, *Ganoderma lucidum* polysaccharides and Boric acid can effectively reduce the emission of toxic and harmful substances in the feces and urine of fatteners. Moreover, it is not known whether the combined addition of Boric acid and plant polysaccharides can have a more effective effect on the production performance, digestive function, antioxidant function and immune function of fatteners, which hurts the scientific and rational use of these additives in pig production and the improvement of breeding efficiency. Therefore, we investigated the effects of the combined addition of Boric acid and plant polysaccharides on the production performance, digestive enzyme activity, immune and antioxidant functions, as well as harmful gas and heavy metal emissions in feces and urine of fatteners, to determine whether the combined addition can further improve the application effect and reduce the emission of harmful substances in pig feces and urine, thereby achieving better overall results.

## 2. Materials and Methods

The experimental protocol was reviewed and approved by the Animal Care and Use Committee of Anhui Science and Technology University.

### 2.1. Experimental Products

In this study, boric acid (analytical purity, 99.5% purity) is provided by China National Pharmaceutical Group Chemical Reagent Co., Ltd. (Shanghai, China) *Astragalus* polysaccharides, *Ganoderma lucidum* polysaccharides (brownish yellow powder) and *Echinacea* polysaccharides have purities of 50%, 70% and 10%, respectively, and provided by Xi’an Aosai Biotechnology Co., Ltd. (Xi’an, China) and Ningshan Guosheng Biotechnology Co., Ltd. (Shanxi, China).

### 2.2. Animals, Dietary Treatments and Experimental Design

A total of 90 crossbred fatteners (Duroc × Landrace × Yorkshire, 90 days old, from the same farm origin) with an initial body weight (IBW) of 41.25 ± 3.07 kg, were evenly divided into five groups, three replicate pens per group and six fatteners per pen. The five treatment groups were as follows: the control group was fed with a basal diet without boric acid and plant polysaccharides (Con), the experimental group I was fed with a basal diet + 40 mg/kg of boric acid (BA) [16], the experimental group II was fed with a basal diet + 40 mg/kg of boric acid + 400 mg/kg of 50% *Astragalus* polysaccharides (BA+APS) [17]; the experimental group III was fed with a basal diet + 40 mg/kg of boric acid + 200 mg/kg of 70% *Ganoderma lucidum* polysaccharides (BA+GLP) [18,19]; the experimental group IV was fed with a basal diet + 40 mg/kg of boric acid + 500 mg/kg of 10% *Echinacea* polysaccharides (BA+EPS) [20]. The whole experiment lasted for 90 days. The basal diet was formulated to meet the nutrient requirements according to the National Research Council (NRC 2012), as shown in Table 1. The basal diet used in the experiment is powder, produced by the feed processing plant of Anhui Hefeng Agriculture and Animal Husbandry Co., Ltd. (Bozhou, China). Before adding to the basal diet, boric acid and plant polysaccharides are weighed separately, first mixed with 1 kg of basal diet, then mixed with 10 kg of basal diet and finally mixed with 100 kg of basal diet.

Pigs were obtained and reared in fattening houses of the Wu Zifeng Experimental Farm at the Anhui Hefeng Agriculture and Animal Husbandry Co., Ltd. and six pigs per pen (18 m^2^ per pen and 3.0 m^2^ per piglet) were raised in the same area for suitable pig density during the whole experiment. Per pig per pen is kept separately with a metal fence on the partially slatted floor, with separate drinking water and feeding trough provided. There was 1 automatic stainless steel nipple drinker and 1 cement feeder (6 slots) per pig. The study was conducted from September to November 2022 in Lixin, Bozhou, Anhui Province. The conditions (temperature, humidity and so on) of the farm were, in real-time, monitored and controlled, and all fatteners were vaccinated following the farm’s routine vaccination program.

### 2.3. Sample Collection

Animals were weighed and labeled before the start of the experiment, and the initial weight measurements were recorded. The per-pen feed intake and uneaten feed were recorded to calculate the average daily feed intake at a fixed time every day. Three days before the end of the experiment, fresh feces and urine were randomly collected from 2 pigs per pen every morning. At the end of the experiment, the pigs were fasted for 12 h, and blood samples were taken from the ear vein, which were stored at 4 °C. The samples were centrifuged at 3000 r/min to obtain serum samples, which were stored at −80 °C. Tissue samples from the duodenum, jejunum, spleen and lymph nodes were collected from pigs after anesthesia [21]. Two pigs per pen were randomly selected for sample collection (30 samples), and the samples were frozen in liquid nitrogen and stored at −80 °C in an ultrafreezer.

### 2.4. Determination of Indices

#### 2.4.1. Growth Performance

Individual pigs were weighed on an empty stomach at 7:00 a.m. on the 28th, 56th and 84th day of the experiment and before slaughter to determine the Average-day-gain (ADG). In addition, the amount of feed provided, uneaten feed and feed intake per pig per day were used to determine the average daily feed intake (ADFI), and gain-to-feed ratio (G/F) of the pig during the developmental stage.

#### 2.4.2. Determination of Intestinal Digestive Enzyme Activity

The thawed duodenum and jejunum tissues was weighed on an electronic balance (accuracy up to 1000th of a gram), homogenized in an ice bath and centrifuged at 3000 revolutions per minute (rpm) for 15 min at 4 °C. The supernatants were obtained and stored at −80 °C for later use. The total protein content in the duodenum and jejunum homogenates was determined using the Total Protein Assay Kit (Nanjing Jiancheng Bioengineering Institute, Nanjing, China). The activities of trypsin, maltase and lipase were measured with the corresponding Enzyme Activity Detection Kits as per the instructions of the manufacturer (Nanjing Jiancheng Bioengineering Institute).

#### 2.4.3. Determination of Cytokine Contents

After thawing the supernatants of spleen and lymph node tissue at room temperature, tumor necrosis factor (TNF-α), interleukin-2 (IL-2), interleukin-6 (IL-6) and interferon-gamma (IFN-γ) in serum samples, spleen and lymph node tissues were measured. The measurement was performed according to the manufacturer’s instructions for the kit (Beijing Dacome Technology Co., Beijing, China).

#### 2.4.4. Determination of Antioxidant Function

After thawing the supernatants of spleen and lymph node tissue at room temperature, the content or activity of malondialdehyde (MDA), superoxide dismutase (T-SOD), glutathione peroxidase (GSH-Px) and total antioxidant capacity (T-AOC) in the spleen and lymph node were measured using Antioxidant Function Assay Kit based on the manufacturer’s instructions (Nanjing Jiancheng Bioengineering Institute).

#### 2.4.5. Determination of Harmful Gases and Metallic Elements in Faeces

Freshly collected 50 g feces and 50 g urine are mixed in an 845 mL plastic bottle for fermentation at room temperature (20 °C) for 24 h. Gas emissions of NH_3_ and H_2_S from feces and urine samples were continuously measured for three days. A portable gas sampling pump (ZC500; suction flow rate at 500 mL/min; Henan Kailu Electronic, Anyang, China) was used to extract gas from 2 cm above the mixture of feces and urine through a small hole at the bottle mouth. The gas values were recorded by the composite multi-gas detector (K-400A; Henan Kailu Electronic Co., Ltd.) [22].

The detection of metal ions and NH_3_-N contents in feces was performed by Qingdao Science and Technology Innovation Quality Testing Co., Ltd. (Qingdao, China)

### 2.5. Statistical Analysis

The normality of the distribution of the data was analyzed using Kolmogorov–Smirnov and the Shapiro–Wilk test (*p* > 0.05). Data following a normal parametric distribution underwent analysis of variance using the General Linear Model (GLM), with the model considering block, time and treatment effects. Tukey’s post hoc test was employed to analyze the significant differences between treatment groups. A *p*-value less than 0.05 was considered statistically significant. Non-normally distributed data were analyzed using the non-parametric Kruskal–Wallis test, the significance level of *p* < 0.05 was considered statistically significant. Designating treatment as the fixed factor and per pen as the experimental units. The dosage and type of additives are the main factors. The initial body weight was included as a covariate in the growth performance analysis. The statistical analyses were conducted using the IBM SPSS Statistics 23.0 software (SPSS Inc., Chicago, IL, USA). Correlations were assessed using Pearson correlation analysis of the Euclidean distance. All the data were presented as mean ± SD.

## 3. Results

### 3.1. Effects of Plant Polysaccharides Combined with Boric Acid on Growth Performance of Fatteners

**Figure 1:**
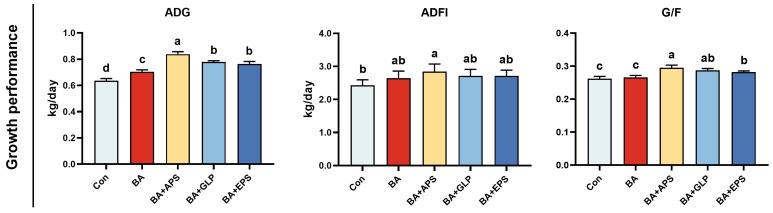
Effects of adding different plant polysaccharides combined with boric acid in the diet on growth performance of fatteners (*n* = 3): ADG: Average daily weight gain; ADFI: Average daily feed intake; G/F: Gain-to-feed ratios. Con: Control; AB: Boric acid; APS: *Astragalus* polysaccharides; GLP: *Ganoderma lucidum* polysaccharides; EPS: *Echinacea* polysaccharides. ^a,b,c,d^ Different letters indicate differences (*p* < 0.05).

Compared to the Con (Figure 1), the ADG was increased in BA, BA+APS, BA+GLP and BA+EPS (*p =* 0.042, *p* = 0.011, *p* = 0.022, *p* = 0.027), the ADFI was increased in BA+APS (*p* = 0.033) and the G/F were also increased in BA+APS, BA+GLP and BA+EPS (*p* = 0.031, *p* = 0.041, *p* = 0.046). Compared with BA, the ADG were increased in BA+APS, BA+GLP and BA+EPS (*p* = 0.025, *p* = 0.0342, *p* = 0.044), and the G/F were also increased in BA+APS, BA+GLP and BA+EPS (*p* = 0.031, *p* = 0.039, *p* = 0.046).

### 3.2. Effects of Plant Polysaccharides Combined with Boric Acid on Digestive Enzyme Activities of Fatteners

**Figure 2:**
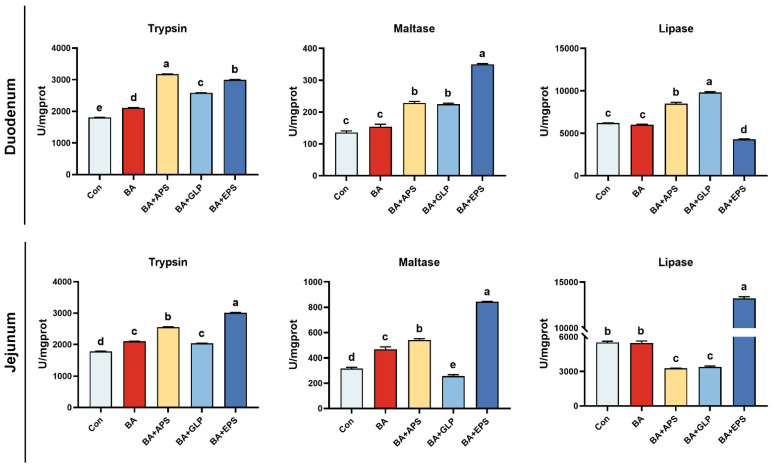
Effects of Adding different plant polysaccharides combined with boric acid in the diet on intestinal digestive enzyme activities of fatteners (*n* = 6): Con: Control; AB: Boric acid; APS: *Astragalus* polysaccharides; GLP: *Ganoderma lucidum* polysaccharides; EPS: *Echinacea* polysaccharides. ^a,b,c,d,e^ Different letters indicate differences (*p* < 0.05).

Compared with the Con (Figure 2), the trypsin activities were increased in the duodenum of fatteners in other groups (*p* = 0.039, *p* = 0.002, *p* = 0.023, *p* = 0.011), and increased in the jejunum of fatteners in other groups (*p* = 0.027, *p* = 0.014, *p* = 0.040, *p* = 0.005). Compared with the Con, the maltase activities were increased by 68.16%, 65.68% and 157.54% in the duodenum of fatteners in the BA+APS, BA+GLP and BA+EPS, respectively (*p* = 0.021, *p* = 0.020, *p* = 0.002), and also increased by 47.19%, 70.05% and 166.36% in the jejunum of fatteners in the BA, BA+APS and BA+EPS (*p* = 0.025, *p* = 0.014, *p* = 0.005), respectively, but decreased in the jejunum of fatteners in the BA+GLP (*p* = 0.034). Compared with the Con, the lipase activities were increased in the duodenum of fatteners in the BA+APS and BA+GLP (*p* = 0.021, *p* = 0.012), and also increased in the jejunum of fatteners in the BA+EPS (*p* = 0.011) but decreased in the duodenum of fatteners in the BA+EPS (*p* = 0.019). Compared with the BA, the trypsin activities were increased in the duodenum of fatteners in the BA+APS, BA+GLP and BA+EPS (*p* = 0.007, *p* = 0.027, *p* = 0.018), and also increased in the jejunum of fatteners in the BA+APS and BA+EPS (*p* = 0.021, *p* = 0.010). Compared with the BA, the maltase activities were increased in the duodenum of fatteners in the BA+APS, BA+GLP and BA+EPS (*p* = 0.024, *p* = 0.027, *p* = 0.007), and also increased in the jejunum of fatteners in the BA+APS and BA+EPS (*p* = 0.038, *p* = 0.011). Compared with the BA, the lipase activities were increased in the duodenum of fatteners in the BA+APS and BA+GLP (*p* = 0.019, *p* = 0.010), and also increased in the jejunum fatteners in the BA+EPS (*p* = 0.019, *p* = 0.010).

### 3.3. Effects of Plant Polysaccharides Combined with Boric Acid on Immune Function of Fatteners

**Figure 3:**
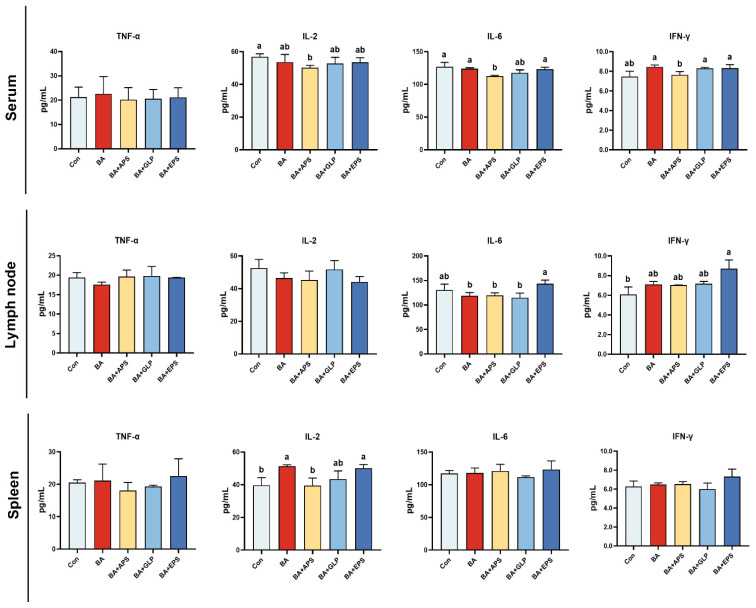
Effects of Adding different plant polysaccharides combined with boric acid in the diet on cytokines of fatteners (*n* = 6): TNF-α: Tumor necrosis factor; IL-2: Interleukin-2; IL-6: Interleukin-6; IFN-γ: Interferon-gamma; Con: Control; AB: Boric acid; APS: *Astragalus* polysaccharides; GLP: *Ganoderma lucidum* poly-saccharides; EPS: *Echinacea* polysaccharides. ^a,b^ Different letters indicate differences (*p* < 0.05).

Compared with the Con (Figure 3), the IL-2 levels were decreased in the serum of fatteners in the BA+APS (*p* = 0.034) but increased in the spleen of fatteners in the BA and BA+EPS (*p* = 0.028, *p* = 0.041). Compared with the Con, the IL-6 levels were decreased in the serum of fatteners in BA+APS (*p* = 0.032), and the IFN-γ levels were increased in the lymph node of fatteners in the BA+EPS (*p* = 0.033). Compared with the BA, the IL-2 levels were decreased in the spleen of fatteners in the BA+APS (*p* = 0.027), the IFN-γ levels were decreased in the serum of fatteners in the BA+APS (*p* = 0.046) and the IL-6 levels were also decreased in the serum of fatteners in BA+APS (*p* = 0.039).

### 3.4. Effects of Plant Polysaccharides Combined with Boric Acid on Antioxidant Activity of Fatteners

**Figure 4:**
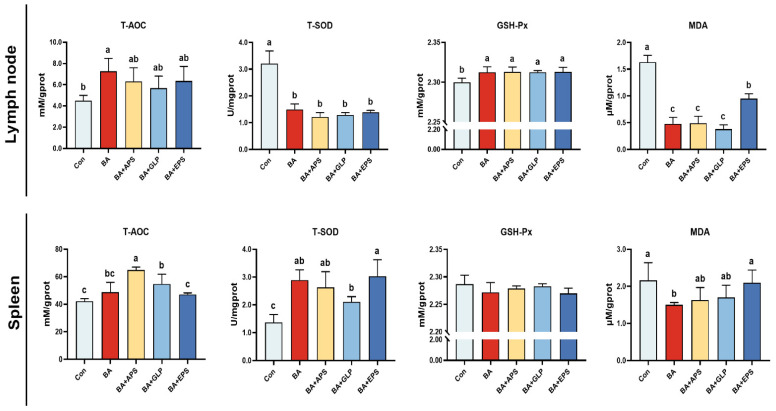
Effects of Adding different plant polysaccharides combined with boric acid in the diet on the antioxidant function of fatteners (*n* = 6): T-AOC: Total antioxidant capacity; T-SOD: Superoxide dismutase; GSH-Px: Glutathione peroxidase; MDA: Malondialdehyde. Con: Control; AB: Boric acid; APS: *Astragalus* polysaccharides; GLP: *Ganoderma lucidum* poly-saccharides; EPS: *Echinacea* polysaccharides. ^a,b,c^ Different letters indicate differences (*p* < 0.05).

Compared with the Con (Figure 4), the T-AOC activities were increased in the lymph node of fatteners in BA (*p* = 0.016) and also increased in the spleen of fatteners in the BA+APS and BA+GLP (*p* = 0.012, *p* = 0.041). Compared with the Con, the T-SOD activities were decreased in the lymph node of fatteners in other groups (*p* < 0.01) but increased in the spleen of fatteners in other groups (*p* = 0.014, *p* = 0.031, *p* = 0.038, *p* = 0.016). Compared with the Con, the GSH-Px contents were increased in the lymph node of fatteners in other groups (*p* < 0.05), and the MDA contents were decreased in the lymph node of fatteners in other groups (*p* < 0.01) and also decreased in the spleen of fatteners in the BA (*p* = 0.026). Compared with the BA, the T-AOC activities were increased in the spleen of fatteners in the BA+APS (*p* = 0.027) and the MDA contents were increased in the lymph node and spleen of fatteners in the BA+EPS (*p* = 0.019, *p* = 0.034).

### 3.5. Effects of Plant Polysaccharides Combined with Boric Acid on the Contents of Harmful Substances in Feces and Urine of Fatteners

**Figure 5:**
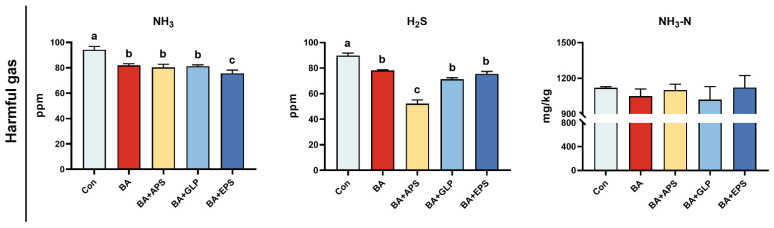
Effects of Adding different plant polysaccharides combined with boric acid in the diet on harmful gas emissions in feces and urine of fatteners (*n* = 6): Con: Control; AB: Boric acid; APS: *Astragalus* polysaccharides; GLP: *Ganoderma lucidum* polysaccharides; EPS: *Echinacea* polysaccharides. ^a,b,c^ Different letters indicate differences (*p* < 0.05).

Compared with the Con (Figure 5), the contents of NH_3_ were decreased in the feces and urine of fatteners in other groups (*p* = 0.024, *p* = 0.026, *p* = 0.024, *p* = 0.012), and the contents of H_2_S were also decreased in the feces and urine of fatteners in other groups (*p* = 0.036, *p* = 0.002, *p* = 0.023, *p* = 0.025). No difference was found in NH_3_-N contents of the feces and urine in other groups. Compared with BA, the contents of NH_3_ were decreased by 9.33% in the feces and urine of fatteners in the BA+EPS (*p* = 0.041), and the contents of H_2_S were also decreased by 50.00% in the feces and urine of fatteners in the BA+APS (*p* = 0.015), while no change in NH_3_-N contents was observed.

**Figure 6:**
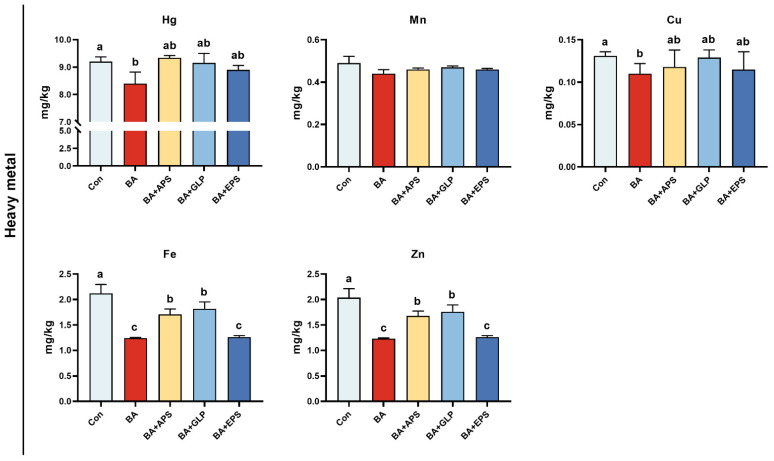
Effects of adding different plant polysaccharides combined with boric acid in the diet on metal elements in feces and urine of fatteners (*n* = 6): Con: Control; AB: Boric acid; APS: *Astragalus* polysaccharides; GLP: *Ganoderma lucidum* poly-saccharides; EPS: *Echinacea* polysaccharides. ^a,b,c^ Different letters indicate differences (*p* < 0.05).

Compared with the Con (Figure 6), no differences were observed in the contents of metal manganese (Mn) in the feces and urine of fatteners in other groups, but the contents of hydrargyrum (Hg), copper (Cu), ferrum (Fe) and zinc (Zn) were decreased in the feces and urine of fatteners in the BA (*p* = 0.036, *p* = 0.037, *p* = 0.008, *p* = 0.009), and the contents of Fe were decreased in the feces and urine of fatteners in the BA+APS, BA+GLP and BA+EPS (*p* = 0.028, *p* = 0.035, *p* = 0.008), and the contents of Zn were also decreased in the feces and urine of fatteners in the BA+APS, BA+GLP and BA+EPS (*p* = 0.032, *p* = 0.039, *p* = 0.009). Compared with the BA, the contents of Fe were increased in the feces and urine of fatteners in the BA+APS and BA+GLP (*p* = 0.024, *p* = 0.013), and the contents of Zn were also increased in the feces and urine of fatteners in the BA+APS and BA+GLP (*p* = 0.027, *p* = 0.015).

### 3.6. Correlation Analysis

#### 3.6.1. The Correlation Analyses between Intestinal Digestive Enzymes and the Contents of Harmful Substances

**Figure 7:**
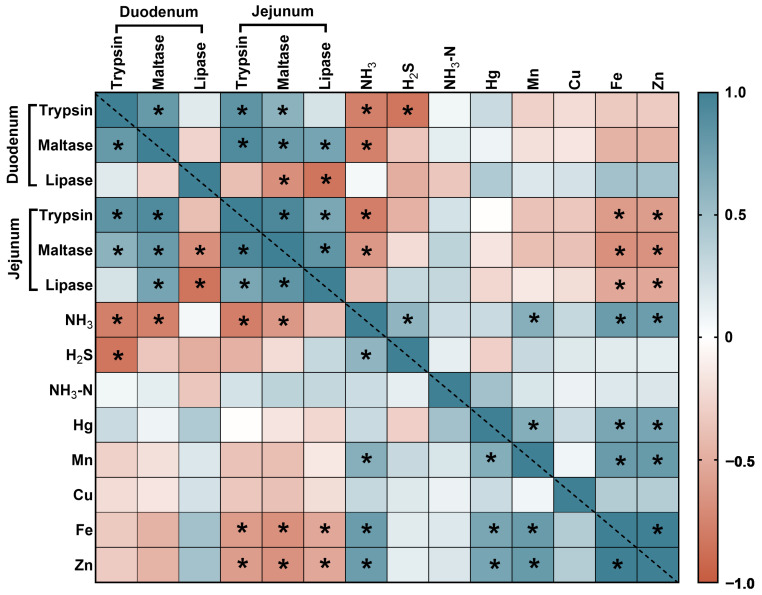
Heatmap describing variables horizontally and vertically for the levels of harmful gases and heavy metals in feces and urine and intestinal digestive enzyme activity. Orange and brown represent a significant positive correlation, blue and light blue represent a significant negative correlation and white represents no correlation. * Indicates *p* < 0.05.

In the duodenum, the intestinal digestive enzymes were significantly negatively correlated with NH_3_ and H_2_S (Figure 7). In the jejunum, the intestinal digestive enzymes were significantly negatively correlated with NH_3_, Fe and Zn.

#### 3.6.2. The Correlation Analyses between Serum Immune Proteins and the Contents of Harmful Substances

**Figure 8:**
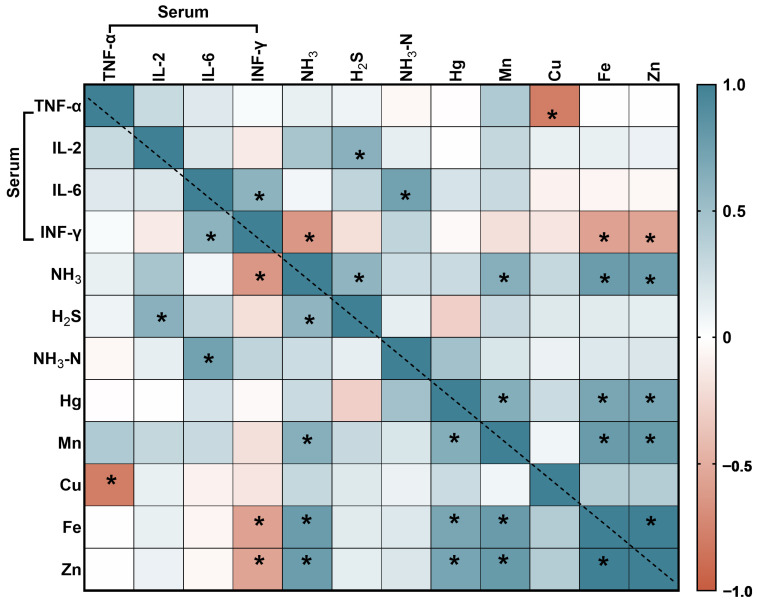
Heatmap describing variables horizontally and vertically for the levels of harmful gases and heavy metals in feces, urine and serum immune proteins. Orange and brown represent a significant positive correlation, blue and light blue represent a significant negative correlation and white represents no correlation. * Indicates *p* < 0.05.

As shown in Figure 8, the IL-2 was significantly positively correlated with H_2_S, the IL-6 was significantly positively correlated with NH_3_-N, the INF-γ was significantly negatively correlated with NH_3_, Fe and Zn, the TNF-α was significantly negatively correlated with Cu.

#### 3.6.3. The Correlation Analyses between Antioxidant Function and the Contents of Harmful Substances

**Figure 9:**
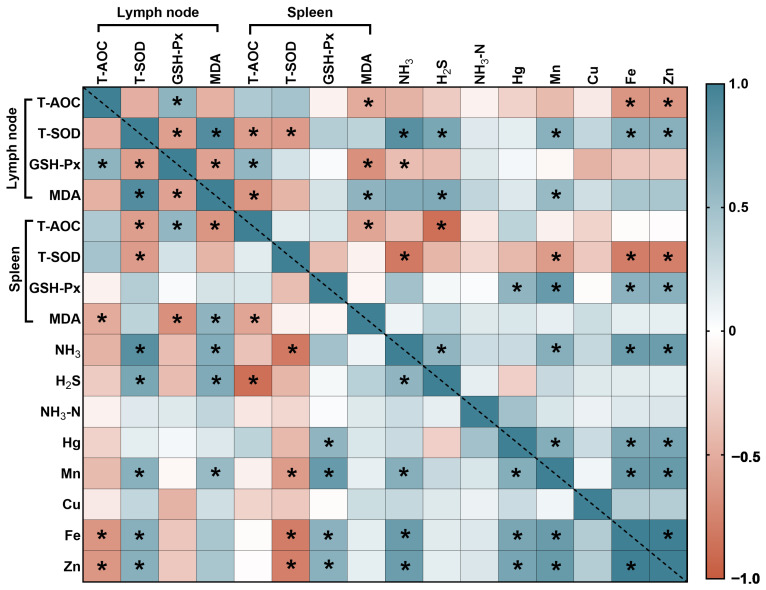
Heatmap describing variables horizontally and vertically for the levels of harmful gases and heavy metals in feces and urine and antioxidant function. Orange and brown represent a significant positive correlation, blue and light blue represent a significant negative correlation and white represents no correlation. * Indicates *p* < 0.05.

In the lymph node, the T-AOC was significantly negatively correlated with Fe and Zn (Figure 9). The T-SOD was significantly positively correlated with NH_3_, H_2_S, Mn, Fe and Zn. The MDA was significantly positively correlated with NH_3_, H_2_S and Mn. In the spleen, the T-AOC was significantly negatively correlated with H_2_S. The T-SOD was significantly negatively correlated with NH_3_, Mn, Fe and Zn. The GSH-Px was significantly positively correlated with Hg, Mn, Fe and Zn. The NH_3_ was significantly positively correlated with Mn, Fe and Zn.

The results of correlation analysis support the hypothesis that boric acid and plant polysaccharides have a significant correlation (*p* < 0.05 or *p* < 0.01) with intestinal digestive enzymes, serum cytokines and antioxidant indicators in the process of dealing with harmful gas and heavy metal emissions in the health fatteners. It may be that the response of intestinal digestive enzymes promotes the body to digest and absorb nutrients, improves the immune function of the body, increases the utilization rate of animal feed and reduces the emission of harmful gases and the emission of feces containing various heavy metal elements.

## 4. Discussion

Plant polysaccharides have various biological activities, which can regulate animal nutritional requirements and potentially improve their digestive capacity, thereby effectively reducing the use of antibiotics in animal husbandry [23]. Studies have shown that the feed supplemented with *Astragalus* polysaccharides effectively enhanced their immunity and growth performance in broiler chickens [24]. Similarly, compared to the control group, fed *Echinacea* polysaccharides increased weight gain and feed conversion ratio during the growing period of broiler chickens [25]. Additionally, it has been shown that providing an appropriate amount of boric acid can increase ADG and ADFI during the growth period of pigs [26].

The functioning of digestive enzymes in the animal intestine is crucial for breaking down and absorbing ingested nutrients. Supplementing exogenous digestive enzymes in the diet can improve animal production performance, feed conversion rate and digestion rate, thereby enhancing intestinal immune function [27] and promoting animal health. Luo et al. found that adding protease and glucoamylase to the diet of broiler chickens can significantly increase trypsin activity, increase digestion emptying time and reduce FCR (F/G) and visible water in feces, thereby alleviating diarrhea caused by newly harvested corn diets [28]. Yi et al. reported that adding enzyme complexes containing amylase, protease and xylanase to the diet can improve nutrient digestibility, increase the concentration of volatile fatty acids and the proportion of Escherichia coli, thereby promoting intestinal health and improve piglet production performance [29]. Previous studies have fully confirmed that increasing the activity of digestive enzymes can promote the absorption of nutrients, increase nutrient digestibility, enhance immune function and thereby enhance animal production performance, promoting body health. However, the secretion and activity of these digestive enzymes can be influenced by various factors, such as the parts of the intestine and cells, the amount and composition of amino acids and the digestion products of ingested proteins [30,31]. Previous studies have shown that supplementation with *Astragalus* polysaccharides in feed can effectively enhance digestive enzyme activity and improve secretion in the chicken intestine [32]. Additionally, it has been reported that feeding *Ganoderma lucidum* polysaccharides to freshwater shrimp (*Macrobrachium rosenbergii*) promoted growth and digestive enzyme activity, thus promoting digestive function [33].

In this study, the combination of different plant polysaccharides and boric acid can enhance the activities of trypsin, maltase and lipase in the duodenum and jejunum of fatteners when compared to the control group and the group supplemented with only boric acid. These results suggest that the combination of different plant polysaccharides and boric acid can improve the ability of the animals to chemically digest nutrients in the intestine. One possible mechanism is that *Astragalus* polysaccharides and *Echinacea* extract can improve the balance of intestinal micro-organisms, thus maintaining intestinal function and promoting overall growth performance and health levels [34,35].

Several studies have highlighted that *Echinacea* can stimulate T-cell phagocytosis and enhance lymphocyte activity in broilers, as well as improve cell antioxidant, anti-inflammatory and antimicrobial activities [36]. Therefore, *Echinacea* can be considered a recommended alternative to antibiotics [37,38,39]. Moreover, *Echinacea* polysaccharides have been shown to improve immune indices and increase levels of INF-α, IFN-γ and IL-2 in sera [40], as well as an increase in the total levels of lgG and γ-interferon along with an increase of important cytokine genes expression in calves [41]. Similarly, feeding *Astragalus* polysaccharides to mice with lung cancer resulted in improvements in white blood cell count, thymus index, spleen index and cytokine levels, thus effectively regulating immune function [42]. Moreover, *Ganoderma lucidum* polysaccharides have been shown to produce immunomodulatory effects by increasing the levels of serum IL-2, TNF-α and IFN-γ, then enhancing the activity of NK cells and T-cells and regulating the immune response [43].

Immune function and spleen lymphocyte proliferation were improved by administering 0.4 mmol/L of boron in mice [10]. The beneficial effects of the combinations of plant polysaccharides and boric acid were observed possibly due to the effect of individual immune-enhancing effects of boric acid in this study because its addition produced a more pronounced improvement in antibody levels and cytokine production in fatteners.

Research focused on the antioxidant function of *Echinacea* has revealed that the addition of 0.5–2.0% *Echinacea* into animal feed could increase the Trolox equivalent antioxidant capacity, as well as catalase (CAT) and SOD activity in the serum and spleen of broilers [26]. Similarly, adding *Echinacea* into the diet of crucian carp could stimulate their growth performance and elicit an antioxidant response [44]. Feeding a large yellow croaker with 150 mg/kg of *Astragalus* polysaccharides increased liver T-AOC, GSH-Px and lysozyme activities [45]. Furthermore, supplementation with *Ganoderma lucidum* polysaccharides has been shown to alleviate oxidative stress and inflammation in rats by up-regulating SOD, CAT and GSH-Px contents, increasing IL-10 levels and preventing excess production of MDA [46]. The addition of 160 mg/kg of boric acid positively affected the development of ostrich kidneys, inhibited cell apoptosis, regulated enzyme activity, improved the antioxidant system and enhanced overall antioxidant capacity [47]. This enhanced antioxidant effect can be attributed to the antioxidant properties of *Astragalus* polysaccharides, *Ganoderma lucidum* polysaccharides and *Echinacea* extract individually. When combined with boric acid, their effects are synergistically enhanced, further promoting their effects.

Animal feces can produce harmful gases, such as NH_3_ and H_2_S. If harmful gases are emitted excessively, they will pollute the air and threaten human health. NH_3_ can cause respiratory diseases, while H_2_S can damage the nervous system [48]. Plant secondary metabolites have been shown to improve the composition of animal rumen bacterial communities, leading to a reduction in methane emissions and influencing rumen fermentation [49]. Previous studies showed that adding a mixture of plant extracts can reduce the emissions of harmful gases (NH_3_ and H_2_S) in the feces of growing and fatteners [50].

In this study, the addition of boric acid alone decreased the contents of NH_3_ and H_2_S. However, the combined use of boric acid and plant polysaccharides further reduced NH_3_ and H_2_S emissions. When boric acid was combined with *Echinacea* polysaccharides, the NH_3_ contents were decreased, while its combination with *Astragalus* polysaccharides led to a reduction of H_2_S contents. The results can be attributed to that boric acid can improve animal digestion and promote protein absorption in the feed, while plant polysaccharides enhance the intestinal microbial community and improve intestinal function [51]. The addition of polysaccharides can regulate rumen microbial populations, promoting the growth of beneficial micro-organisms while inhibiting methanogenic bacteria, thus helping to reduce methane production [52,53]. Thus, the combined supplementation of boric acid and plant polysaccharides enhances gastrointestinal digestion, leading to a reduction in the emission of harmful gases in feces.

Excessive emissions of heavy metal substances from feces can have detrimental effects on the environment and food safety. Moreover, the long-term accumulation of metals in the soil can lead to risks for both the atmosphere and agricultural products Common metal residues found in livestock and poultry feces include Zn, Fe, lead, chromium, cadmium, arsenic, Hg and others. In this study, the addition of boric acid alone led to a reduction of contents of Cu, Fe and Zn, when compared to the control group. However, when boric acid was combined with plant polysaccharides—particularly *Echinacea* polysaccharides—further reductions in the contents of Fe and Zn were noted. The reason may be that a series of effects of boron on the metabolism of minerals in animal organisms [54], at the same time, the addition of polysaccharides may also have a series of beneficial effects on the absorption and utilization of minerals by the body [55]. By combining the two, the body can enhance its absorption and utilization of minerals, thereby reducing the excretion of heavy metals through feces and urine [56].

As one of the most common substrates in biogas production, cow and pig faces are often considered to provide necessary nutrients and trace elements to stabilize the biogas process [57], and the harmful gases can have a huge impact on the environment and seriously pollute human health. Research reports indicate that supplementing with lipase in diet can promote the digestion rate of nutrients, thereby having a beneficial effect on intestinal proteases and reducing NH_3_ emissions in pigs. This is consistent with our research findings, which indicate that NH3 decreases with the increase of digestive enzymes [58]. When livestock faces are used as fertilizer, metal elements present as common pollutants in livestock manure may cause terrestrial ecological toxicity. Research has found that zinc and copper are the main factors affecting the overall soil quality [59]. Research has reported that there is a correlation between Fe, Mn and Al elements in the body and the inflammatory response in humans and animals [60], which is consistent with this conclusion. Previous results showed that a diet with a copper level of 20 mg·kg^−1^ significantly increased TNF-α of serum in piglets [61,62]. This is consistent with our results. Further studies have pointed out that heavy metals or toxic elements are one of the interfering factors affecting the absorption process of trace elements from the intestine [63].

This study analyzed the correlation between the harmful gas and element content and intestinal digestive enzymes, serum immune proteins and antioxidant function. It was found that intestinal digestive enzyme activity, serum cytokines and antioxidant function showed a certain correlation with the contents of harmful gases, which may affect the emission of harmful gases and heavy metals by animals, thereby reducing their environmental pollution.

However, in our experiment, choosing fattening pigs as the research object has certain limitations. Different animals have different sensitivities to the added doses of boron and plant polysaccharides [64,65]. Broilers and ostriches have significantly different sensitivities to boron compared to rats. Broilers and ostriches need to supplement higher doses of boron to have beneficial effects. Moreover, environmental conditions have a direct impact on the application effect of boron and plant polysaccharides. Therefore, further research is needed to confirm the application effect of the results of this study in other animals.

## 5. Conclusions

In this study, the combined use of boric acid and plant polysaccharides in the diets of fatteners led to favorable effects, when compared to the use of boric acid alone. In particular, the combined use of boric acid and plant polysaccharides increased ADG and G/F and increased the trypsin activity in the duodenum and jejunum and GSH-Px content of lymph nodes, but decreased MDA content in lymph nodes. So, the combination of boric acid and plant polysaccharides can improve the digestion and absorption of nutrients by enhancing intestinal digestive enzyme activity and antioxidant function. With the increase of digestive enzyme activity, the body can effectively utilize various nutrients, which can reduce the release of harmful gases such as NH_3_ and H_2_S and reduce the content of harmful heavy metals in feces and urine. Based on the above results, the combination of boric acid and *Echinacea* polysaccharides presented the most pronounced benefits. This study provided a basis for future research on boric acid and plant polysaccharides as a feed additive in pigs.

## Figures and Tables

**Table 1:** Diet composition and nutrient level of basal diet (air-dried basis).

Ingredients	Content (%)	Nutrients	Content
Corn	25.75	ME, kcal/kg	3055.00
Wheat	24.00	DM, %	87.77
Wheat middlings mix	18.50	CP, %	13.51
Wheat flour	15.00	EE, %	2.97
Rice bran meal	12.50	CF, %	3.68
Limestone (40)	1.03	Ca, %	0.75
Calcium hydrogen phosphate	0.95	Avail P, %	0.30
Soybean oil	0.80	Total AA, %	12.15
Sodium bicarbonate	0.28	Total Lys, %	0.81
NaCl	0.20	Total Met, %	0.25
L-Lysine HCl	0.56		
*L*-threonine	0.10		
Ethoxyquin	0.03		
Vitamins and minerals Premix ^a^	0.30		
Total	100.00		

This feed formula comes from Anhui Hefeng Animal Husbandry Co. Ltd., and the nutritional level is calculated. ME: Metabolizable Energy; DM: Dry Matter (air dried); CP: Crude Protein; EE: Crude Ether Extract; CF: Crude Fiber; Avail P: Available Phosphorus; AA: Amino Acid; Lys: Lysine; Met: Methionine. ^a^ Provided the following per kg of diet: Vitamin A, 12,500 IU; Vitamin D, 1250 IU; Vitamin E, 125 IU; Vitamin B12, 90 µg; Vitamin B2, 10 mg; Pantothenic acid, 48 mg; Niacin, 35 mg; Folic acid, 4.5 mg; Biotin, 0.25 mg; Fe, 130 mg as iron sulfate; Zn, 180 mg as zinc sulfate; Cu, 15 mg as copper sulfate; Mn, 30 mg as manganese sulfate; I, 0.60 mg as calcium iodate; Se, 0.25 mg as selenate.

## Data Availability

Raw data collected and presented in this study are available on request from the corresponding author.
